# Reductive Stability of Organic Dyes in Nanocatalyst-Assisted Sodium Borohydride Systems

**DOI:** 10.1021/acsomega.6c00189

**Published:** 2026-05-05

**Authors:** Rahina Mohammad Kunhi, Jishina Karunakaran, Rashmi Kunhiraman, Manjunatha Pattabi, Nanditha Thayyath Kizhakkeveettil, Rani Manjunatha Pattabi, Gurumurthy Sangam Chandrasekhar

**Affiliations:** 1 Department of Materials Science, 29082 Mangalore University, Mangalagangotri 574199, India; 2 125853 Manipal Institute of Technology, Manipal Academy of Higher Education, Manipal, Karnataka 576104, India

## Abstract

Catalytic reduction of organic dyes using nanocatalysts and sodium borohydride (NaBH_4_) is widely regarded as an effective strategy for wastewater remediation. However, key mechanistic details underlying the long-term stability of dye removal have remained inadequately addressed. This work provides a systematic comparative investigation of the catalytic reduction behaviors of cationic dyes (methylene blue and rhodamine B) and anionic dyes (methyl orange and congo red) under nanoparticle catalysis with controlled atmospheric conditions. Our results demonstrate that cationic dyes undergo a reversible transition to colorless forms via NaBH_4_ reduction but are reoxidized back to their original-colored state upon exposure to atmospheric oxygen or hydrogen peroxide, confirming the nonpermanent nature of dye removal. In contrast, anionic dyes exhibit irreversible reduction, with decolorization persisting even after prolonged oxidant exposure. These findings, supported by UV–visible and Fourier-transform infrared spectroscopy, reveal critical roles of molecular structure and functional groups in dictating dye redox stability. By explicitly differentiating mechanistic pathways for cationic and anionic dyes, this study provides a new framework for evaluating nanocatalyst-assisted dye remediation. These insights underscore the need for rigorous mechanistic assessment of catalytic dye treatment strategies in environmental applications.

## Graphical abstract

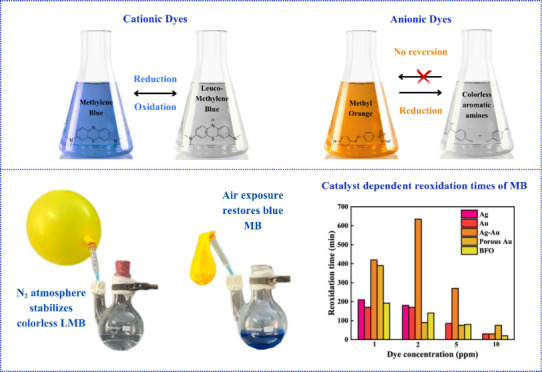

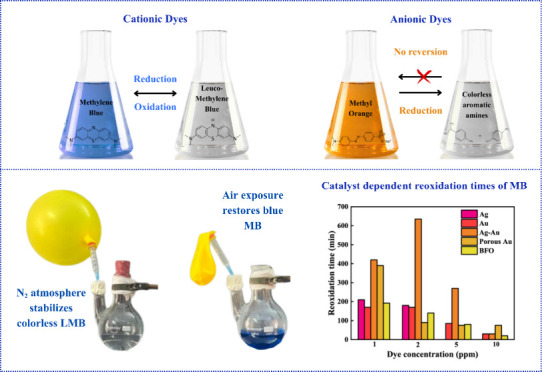

## 1 Introduction

Organic dyes, widely used in industries like textiles, paper, and plastics, pose significant environmental concerns due to their toxicity, nonbiodegradability, and water contamination risks. Conventional dye removal techniques, including membrane filtration, adsorption, and oxidation processes, are often limited by high operational costs, substantial energy demands, and the generation of toxic byproducts, thereby necessitating the development of more sustainable and efficient alternatives.^1–6^ In this context, supramolecular approaches have emerged as promising strategies, offering a robust mechanistic framework for the selective adsorption-based removal of cationic and anionic dyes on catalyst surfaces through precisely tailored intermolecular interactions.^7,8^ Notably, dye adsorption capacity is significantly enhanced on multivalent hydrophilic surfaces with selectivity governed by surface recognition processes arising from cooperative multivalent supramolecular interactions, including classical synthons such as pyridine–carboxylic acid, O–H···N hydrogen bonding, cation−π interaction, π–π stacking, and electrostatic interactions.^7,8^ Complementing these adsorption-driven strategies, nanoparticle-catalyzed reduction using NaBH_4_ has been widely adopted for dye degradation, leveraging the high surface area and catalytic activity of metallic (Au, Ag, Cu, Fe, Pt, Pd) and nonmetallic nanoparticles.^2,3,9–35^ In this process, NaBH_4_ acts as a potent reducing agent, donating electrons to dye molecules via nanoparticle-mediated electron transfer, wherein the nanoparticles function as efficient electron relays, thereby facilitating rapid and effective dye decolorization.^36^ Dye decolorization in the presence of NaBH_4_ and nanocatalysts does not necessarily indicate complete mineralization to carbon dioxide and water, contrary to common assumptions.^31,37^ Instead, the process typically involves the reduction of dye molecules to their corresponding leuco (reduced) forms.^31^ These reduced intermediates are generally unstable and readily revert back to the original dye structure. Such reversible redox transformations are characteristic of clock reactions, which are based on oxidation–reduction mechanisms and are often accompanied by a visually observable color change.^17,31^ This phenomenon raises critical questions about the true efficacy of catalytic reduction for dye remediation.

During our investigations into the catalytic decolorization of organic dyes, we observed a substantial difference in the reduction behavior of cationic versus anionic dyes mediated by nanocatalysts in the presence of NaBH_4_. Notably, cationic dyes such as methylene blue (MB) and rhodamine B (RhB) reverted to their original-colored forms over time, while anionic dyes like methyl orange (MO) and congo red (CR) remained in their reduced, colorless state for extended durations even upon prolonged exposure to atmospheric oxygen. An extensive literature review indicates that this distinct charge-dependent reversibility in catalytic dye reduction has not been reported to date. Accordingly, this study provides a comprehensive examination of catalytic reduction for representative cationic (MB, RhB) and anionic (MO, CR) dyes using a variety of nanocatalysts including metallic (Au, Ag, Ag–Au bimetallic, Porous Au) and the oxide-based bismuth ferrite (BFO) nanoparticles (NPs) under carefully controlled experimental conditions. By conducting reduction experiments under inert atmosphere (N_2_) and oxidative conditions (H_2_O_2_), and systematically varying parameters such as dye concentration, catalyst loading, NaBH_4_ concentration, and pH, this work elucidates the influence of molecular structure and reaction environment on dye redox stability. These insights provide crucial mechanistic understanding necessary for advancing catalytic dye degradation technologies in environmental applications.

## 2 Experimental Section

### 2.1 Materials and Methods

All chemicals used in this study were of analytical grade and used as received without further purification. Bismuth nitrate (Avra, Hyderabad), iron nitrate (Loba Chemie, Mumbai), ascorbic acid (Fisher Scientific, Mumbai), nitric acid, sodium borohydride (NaBH_4_) (SRL, Maharashtra), chloroauric acid hydrate (HAuCl_4_·xH_2_O) (Spectrochem, Mumbai), silver nitrate (AgNO_3_) (Molychem, Mumbai), and trisodium citrate (Nice Chemicals, Cochin), methyelene blue (Fusion biotech, New Delhi), methyl orange (HiMedia, Thane), rhodamine B (Molychem, Mumbai), congo red (Molychem, Mumbai) were procured from commercial suppliers.

### 2.2 Synthesis of Nanoparticles

#### 2.2.1 Synthesis of Ag Nanoparticles

Ag NPs were synthesized via conventional citrate method. In brief, 50 mL of an aqueous solution of 1 mM AgNO_3_ was brought to a boil, to which 5 mL of 1% trisodium citrate solution was added dropwise with vigorous stirring. The heating was maintained for 15 min, after which the solution was allowed to cool to room temperature with continuous stirring, resulting in the formation of yellow-colored Ag NPs.^38^

#### 2.2.2 Synthesis of Au Nanoparticles

Au NPs were synthesized utilizing the Turkevich method. This procedure employed an aqueous solution of 1 mM HAuCl_4_ as the gold precursor and 1% trisodium citrate as the reducing agent. The precursor solution, measuring 50 mL, was heated to boiling, after which 5 mL of the reducing agent was added. The heating was maintained for 15 min, during which the solution’s color transitioned to pink. Subsequently, the solution was allowed to cool to room temperature with continuous stirring, culminating in the formation of Au NPs.^39^

#### 2.2.3 Synthesis of Ag–Au Bimetallic Nanoparticles

The seed colloidal technique was employed to synthesize Ag–Au bimetallic NPs. In this method, Au NPs synthesized via the Turkevich method were used as the seed solution,^39^ while AgNO_3_ served as the silver precursor and trisodium citrate and ascorbic acid acted as reducing agents. In a typical synthesis, 10 mL of a 25% diluted Au seed solution was stirred, followed by the sequential addition of 1 mL of 1% trisodium citrate and 1.2 mL of 10 mM AgNO_3_. Subsequently, 0.4 mL of 100 mM ascorbic acid was introduced into the reaction mixture. The mixture was maintained under vigorous stirring for 30 min to facilitate uniform nucleation and controlled growth of silver on the gold seeds, ultimately leading to the formation of well-dispersed Ag–Au bimetallic NPs.^40^

#### 2.2.4 Synthesis of Porous Gold (Porous Au) Nanoparticles

Porous Au NPs were synthesized through a transmetalation (TM) process using Ag NPs as the starting material, following the method reported by Van Dong et al.^38^ (Section 2.2.1) i.e., 50 mL of a 1 mM AgNO_3_ solution was brought to boiling, after which 5 mL of 1% trisodium citrate was added under vigorous stirring. The mixture was maintained at boiling for 15 min and then allowed to cool to room temperature while stirring continuously. For the TM reaction, 25 mL of the synthesized Ag nanoparticle solution (diluted to 10%) was mixed with 20 mL of a 0.25 mM Au precursor solution across a chicken egg membrane at room temperature with continuous stirring. The progression of the reaction was monitored by the gradual color change of the solution to sea blue, confirming the formation of porous Au NPs.^41^

#### 2.2.5 Synthesis of BFO Nanoparticles

Nanosized BFO particles were synthesized via the autocombustion method using equimolar concentrations (0.25 M) of bismuth nitrate and iron nitrate as precursors, with ascorbic acid employed as a chelating agent in a 2:1 molar ratio with the metal ions. Initially, equimolar solutions of bismuth nitrate and iron nitrate were prepared in dilute nitric acid and stirred for 15 min to ensure homogeneity. Ascorbic acid was then added dropwise to the mixed solution, which was subsequently heated under vigorous stirring at 110 °C until complete solvent evaporation occurred. The resulting mixture underwent spontaneous autoignition, accompanied by the release of gases, yielding a loose brownish powder. This powder was collected, finely ground, and calcined at 550 °C for 1 h to obtain phase-pure BFO NPs, as previously reported.^42^

### 2.3 Catalytic Reduction of Different Organic Dyes in the Presence of Nanoparticles

The reduction of various dyes (MB, RhB, MO and CR) using different NPs (Ag, Au, Ag–Au, porous Au, and BFO NPs) was studied in the presence of NaBH_4_ at room temperature (∼30 °C). In a typical reaction, 1 mL of Ag NPs, 0.5 mL Ag–Au NPs, 5 mL of porous Au NPs (with catalyst concentration selected based on reduction efficiency, as detailed in Section 4.2.4) or 25 mg of BFO NPs was added to 100 mL of 2 ppm dye solution (20 ppm in case of CR), followed by 1 mL of freshly prepared 0.25 M NaBH_4_ solution. The mixture was thoroughly mixed, at a stirring rate of 10,000 rpm and the absorption was monitored using a UV–vis spectrophotometer.

$$\mathrm{Reduction}/\mathrm{Reoxidation}\left(\%\right)=\frac{A_{0}-A_{t}}{A_{0}}\times 100$$ 1

where A_0_ represents the initial absorbance at t = 0, and A_t_ denotes the absorbance at a given time t during the reduction process.

## 3 Characterization

Structural features and morphologies were examined via field emission scanning electron microscopy (FESEM; Carl ZEISS 03–81). Energy-dispersive X-ray spectrometry (EDS; Oxford Instruments) was used to confirm the stoichiometric ratio. UV–Vis diffuse reflectance spectra (DRS) were recorded at room temperature using a Holmarc UV–Vis NIR spectrophotometer (model HO-SPA-1990P) in the 350–800 nm range with barium sulphate (BaSO_4_) as the reference. The optical properties and absorption spectra for dye degradation were analyzed using a Shimadzu UV–vis spectrophotometer. Functional groups were identified through Fourier Transform Infrared Spectroscopy (FTIR; SHIMADZU IR SPIRIT) with a resolution of 4 cm^–1^ in transmittance mode. For FTIR analysis, the sample was prepared by dissolving the dye in a KBr matrix to form a highly concentrated mixture. The mixture was then thoroughly dried to remove residual moisture and subsequently used for spectral measurements.

## 4 Results and Discussion

### 4.1 Optical, Morphological, and Compositional Characterization of Nanocatalysts

Metal NPs demonstrate enhanced UV–vis absorbance compared to ceramic NPs due to the presence of free conduction electrons, which enable localized surface plasmon resonance at respective wavelength. Enhanced light–matter interaction influenced by structural, compositional, and surrounding mediums make them potential photocatalysts.^43^ Figure 1a–c presents the UV–visible absorption spectra of Au, Ag, Ag–Au, BFO and porous Au nanocatalysts, highlighting the characteristic surface plasmon resonance (SPR) peaks of the metallic NPs and the absorption edge of BFO NPs. The Ag NPs exhibit a distinct SPR band at 423 nm, confirming the formation of stable and homogeneous particles.^44^ The formation of Au NPs is evidenced by a strong SPR characteristic peak at 528 nm, indicative of monodispersed, spherical Au NPs.^45^ Upon incorporation of Ag^+^ ions (1.2 mL of 10 mM AgNO_3_), the SPR band of Au NPs shifts markedly from 528 nm to a sharper and more intense peak at 410 nm, reflecting a blue shift associated with morphological modifications and the controlled reduction of Ag^+^ ions, leading to the formation of an Ag shell over the Au core.^40^

**Figure 1:**
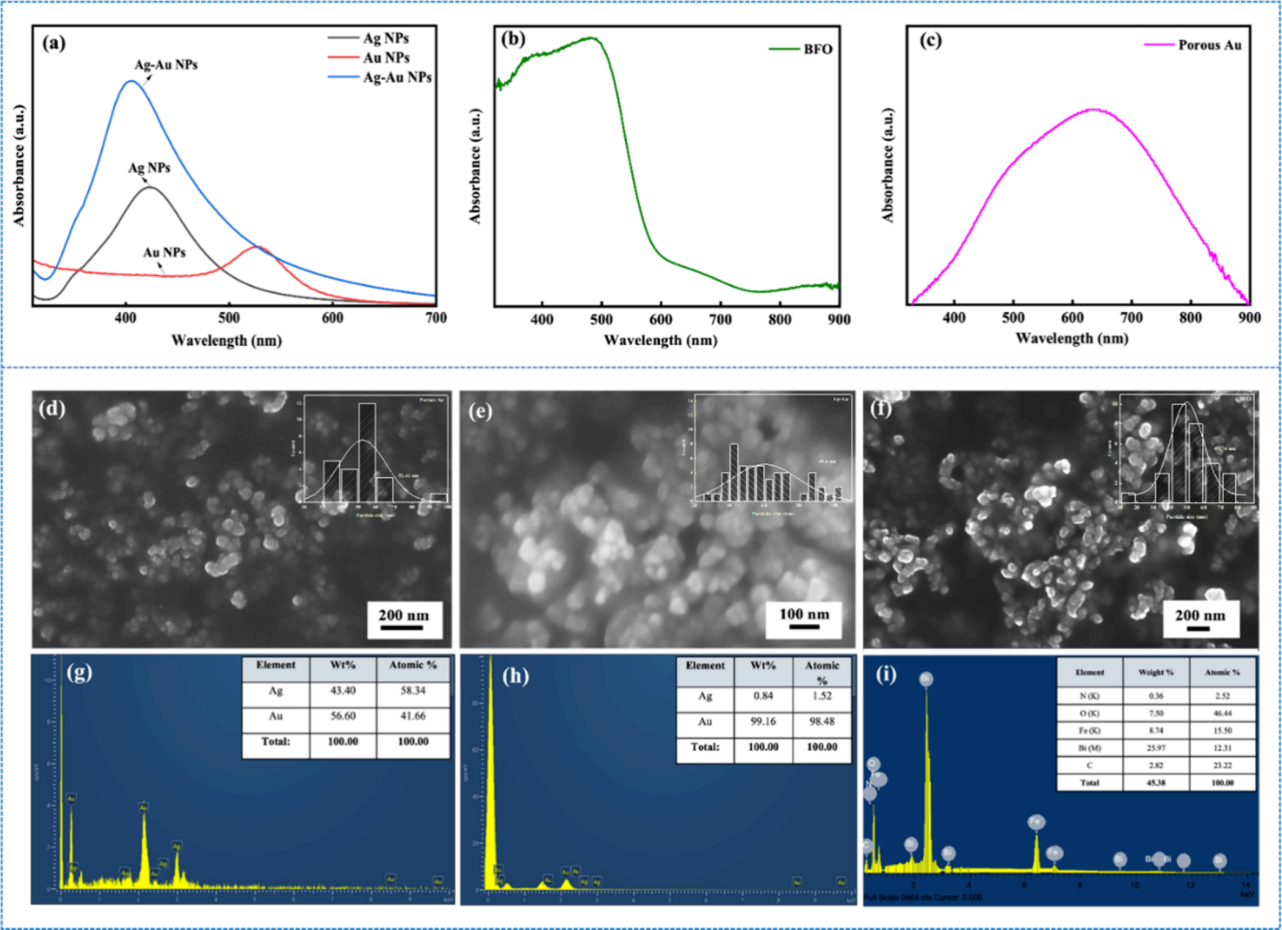
UV–vis absorption spectra of (a) Ag, Au, and Ag–Au NPs, (b) BFO NPs, and (c) porous Au NPs. FESEM images of (d) Ag–Au NPs, (e) porous Au NPs, and (f) BFO NPs, with corresponding EDS spectra of (g) Ag–Au NPs, (h) porous Au NPs, and (i) BFO NPs.

The formation of porous Au NPs is achieved using transmetalation reaction (TM reaction) between sacrificial Ag NPs and Au (III) ions using an eggshell membrane at room temperature. During TM reaction, a progressive damping of the Ag plasmon band is observed, signifying the oxidation and dissolution of Ag atoms. Concurrently, a time-dependent red shift in the absorption maximum indicates an increasing Au contribution, while peak broadening suggests the development of internal voids (Figure S1). This evolution is attributed to the simultaneous dissolution of Ag and deposition of Au, resulting in the formation of a thermodynamically stable porous Au, facilitated by the comparable atomic radii of both metals. The eventual disappearance of the Ag SPR band, along with the emergence of a broad absorption feature, confirms the formation of a thin Au shell via the oxidative reduction of AuCl_4_^–^ ions on sacrificial Ag templates. As the reaction progresses, the appearance of a broad band around 639 nm and its further broadening are associated with decreased wall thickness and dealloying-induced leaching of Ag atoms, which contribute to additional red shifting of the plasmon band.^41^ Furthermore, BFO NPs exhibits visible-light activity with an absorption edge around 587 nm consistent with its narrow band gap.^42^

Figure 1d–f shows the morphology obtained from FESEM, revealing that the synthesized nanocatalysts are homogeneous and nearly spherical. The average particle size was determined using ImageJ software, and the corresponding histogram, representing the statistical distribution of particle sizes. The mean particle sizes were found to be 49.6, 52.42, and 49.76 nm for Ag–Au, porous Au, and BFO NPs, respectively. Additionally, the EDS spectra (Figure 1g–i) confirm the elemental composition of the respective NPs based on their atomic percentages. In the case of BFO NPs, slight variations in the atomic percentages of Bi and Fe observed due to the volatilization of constituent ions during the heat treatment process.^42^

### 4.2 Reduction of Various Organic Dyes

The reduction behavior of organic dyes depends on their structural and chemical properties, including chromophores, absorbance characteristics, and redox potentials, which influence their interactions with reducing agents. Dyes are classified by chemical structure and further subclassified as anionic or cationic, based on ionizable groups in their molecules, which determine their charge in aqueous solutions and reduction behavior with agents like NaBH_4_. Common examples include anionic dyes such as MO and CR `and cationic dyes such as MB and RhB.

The catalytic reduction of different dyes (MB, RhB, MO, and CR) by freshly prepared NaBH_4_ solution was investigated using various nanocatalysts (Ag, Au, Ag–Au, porous Au, and BFO NPs) across a wide pH range (4, 6, 7, 10, 13). In the absence of a nanocatalyst, all four dyes exhibited an immediate increase in absorption peaks, indicating the formation of an intermediate compound (Figure S2 in the Supporting Information). Although slight reduction occurred over time, complete dye reduction was not achieved, highlighting the kinetic limitations of NaBH_4_ despite its thermodynamic favorability.^31,46^ This insufficiency arises from kinetic barriers differences in thermodynamic potentials between NaBH_4_ and the dyes, rendering the reduction process kinetically unfavorable.^20^

When nanocatalysts were introduced into the dye-NaBH_4_ system, the characteristic UV–vis absorption peak of MB (cationic dye) at 664 nm disappeared immediately, with a new peak emerging at 259 nm, corresponding to the leuco-methylene blue (LMB) peak. However, all five colorless solutions gradually reverted to blue over time (Table 1), indicating the reconversion of LMB back to MB (Figure 2). This reverse reaction, although reported by a few researchers^17,31^ has been largely overlooked in previous studies, including us^34,35^ and is often misinterpreted as complete MB degradation.^2,3,12–16,18–28^

**Figure 2:**
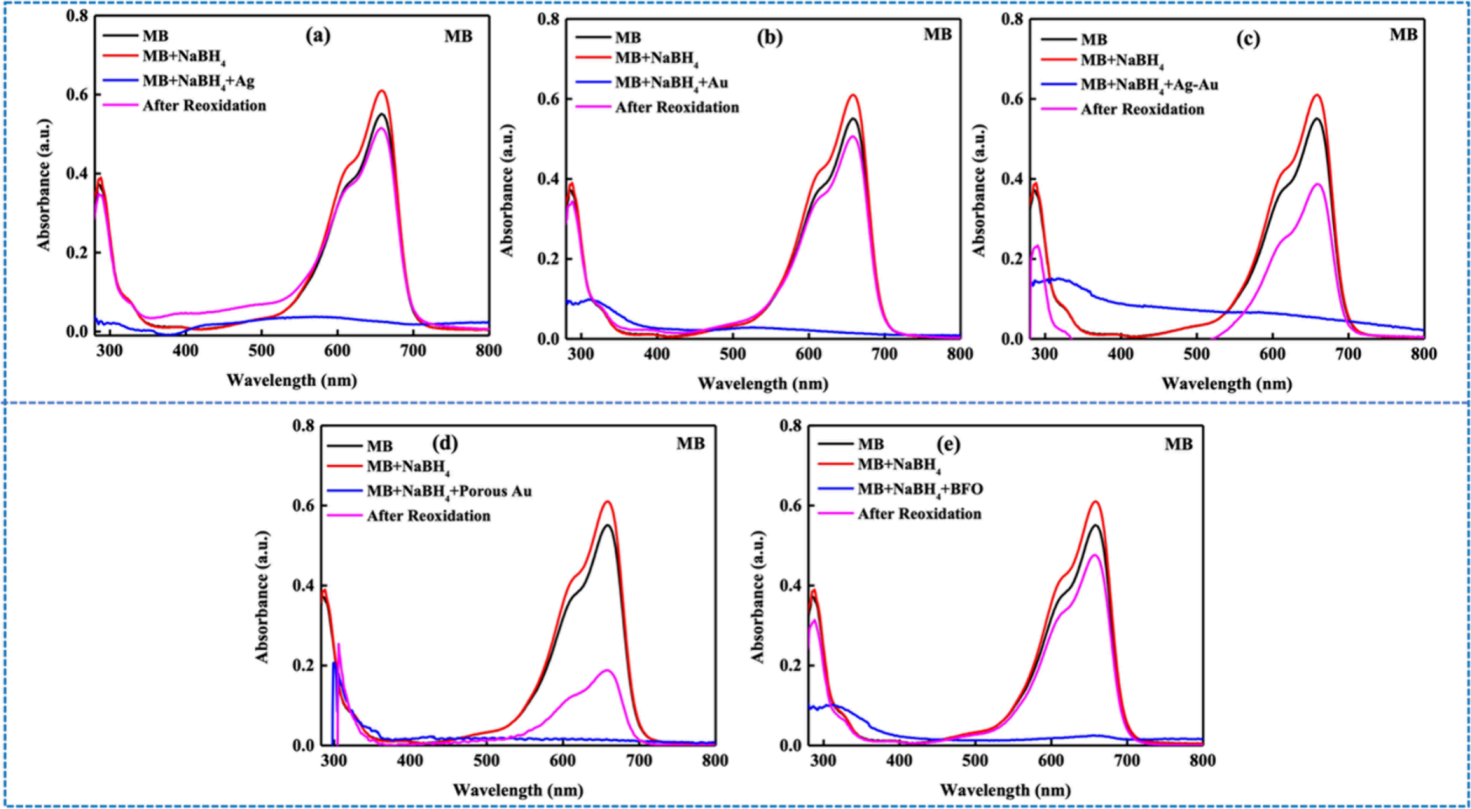
Reduction and reoxidation of MB in the presence of NaBH_4_ and various nanocatalysts, including (a) Ag, (b) Au, (c) Ag–Au, (d) porous Au, and (e) BFO NPs, under ambient conditions and neutral pH.

**Table 1:** Time Taken and Percentage of Reoxidation of MB Dye after Immediate Reduction

nanocatalysts	reoxidation time (min)	% reoxidation
Ag	180	93.5
Au	150	92
Ag–Au	330	86
porous Au	60	34
BFO	160	70

A similar transient reduction was also observed for the cationic dye, RhB. However, although the reduction was immediate in the case of MB, for all the nanocatalyst studied, the absorption peak at 550 nm (λ_max_) for RhB vanished upon nanocatalyst-assisted reduction at varying rates depending on the catalyst, only to gradually reoxidize to its initial state, as confirmed by the reoxidized absorption spectra (Figure 3) and corresponding reoxidation times (Table 2). Notably, such reversible reoxidation behavior for RhB has not been reported in the literature.

**Figure 3:**
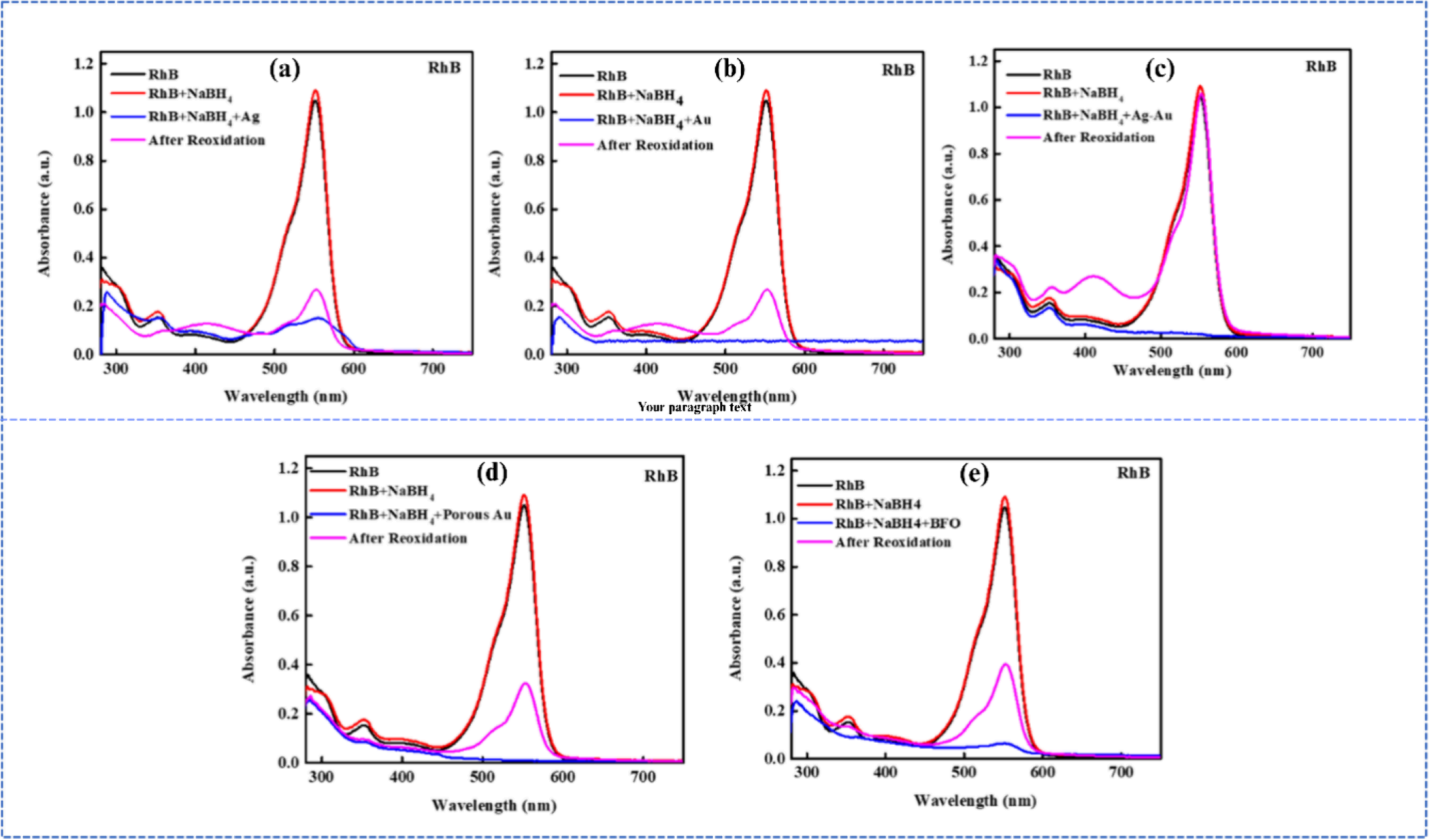
Reduction and reoxidation of RhB in the presence of NaBH_4_ and various nanocatalysts, including (a) Ag, (b) Au, (c) Ag–Au, (d) porous Au, and (e) BFO NPs, under ambient, neutral pH conditions.

**Table 2:** Time Required for Reduction, Reoxidation, and Percentage Reoxidation of RhB Dye

dye	nanocatalyst	reduction time (min)	reoxidation time (min)	reoxidation (%)
RhB	Ag	63	7 h 22 min	25
	Au	0	7 h 55 min	24
	Ag–Au	29	8 h 5 min	100
	porous Au	0	7 h 33 min	31
	BFO	57	7 h 15 min	37

In contrast, the reduction of anionic dyes, such as MO and CR, was permanent. The aqueous solution of MO, initially displaying an orange-red hue with a maximum absorbance at 464 nm, lost its absorption peak immediately upon nanocatalyst addition (Figure 4). There was no reoxidation observed even after prolonged exposure to atmospheric oxygen for a duration of one month.

**Figure 4:**
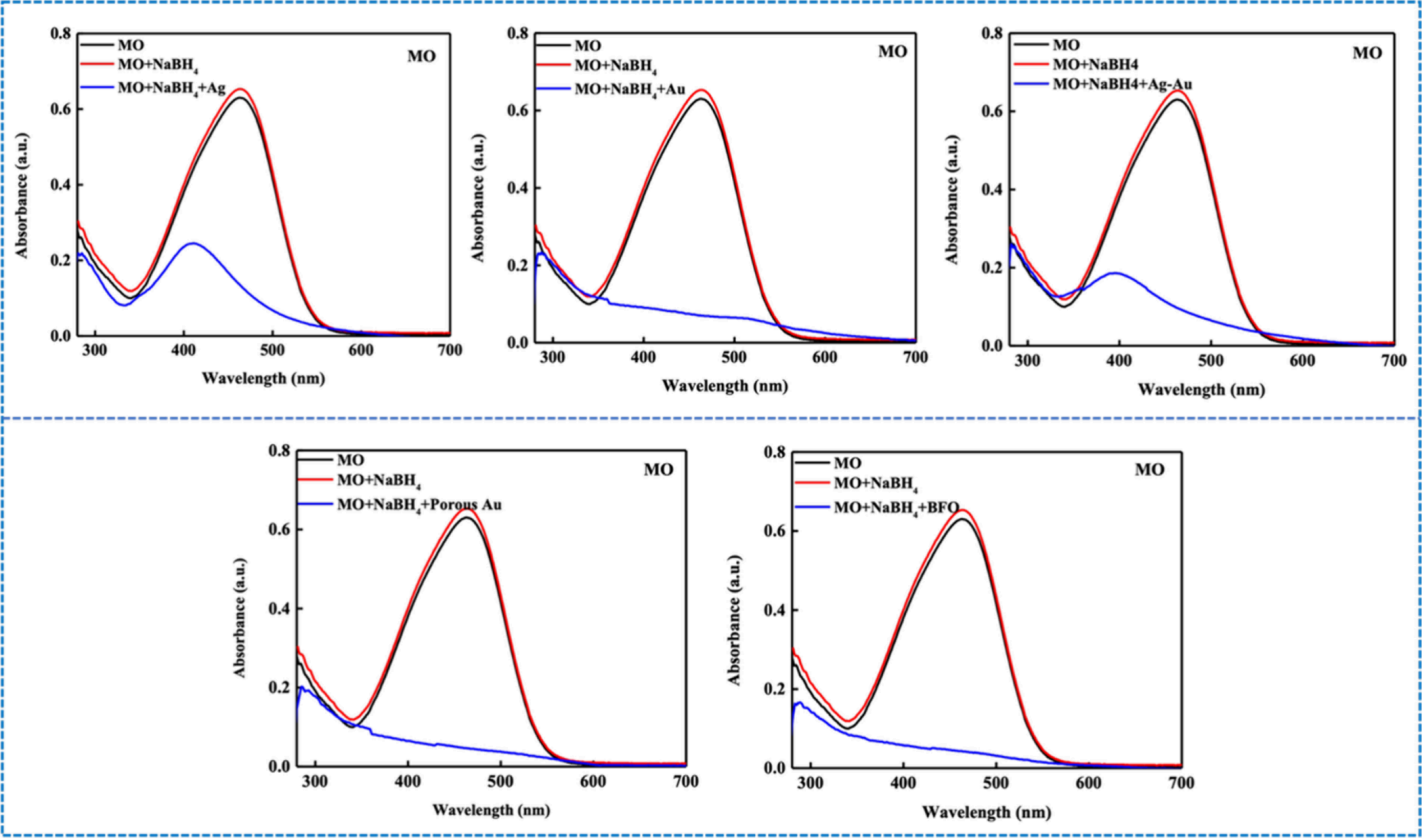
Reduction of MO in the presence of NaBH_4_ and various nanocatalysts, including Ag, Au, Ag–Au, porous Au, and BFO NPs, under ambient, neutral pH conditions.

Another anionic dye, CR, also exhibited similar transformation, with its absorption peak at 499 nm disappearing after nanocatalyst treatment (Figure S3). To improve measurement accuracy, a concentration of 20 ppm of CR was used instead of 2 ppm, as the latter produced absorbance values too low to reliably assess the reduction behavior. Notably, even after a month of exposure to atmospheric oxygen, these anionic dyes did not revert to their original state. Table 3 lists the reduction time for both MO and CR using the various nanocatalysts.

**Table 3:** Time Required for Reduction of MO and CR Dye

dye	nanocatalyst	reduction time (min)	reoxidation time (min)
MO	Ag	immediate	no reoxidation
	Au		
	Ag–Au		
	porous-Au		
	BFO		
CR	Ag	8	no reoxidation
	Au	7	
	Ag–Au	6	
	porous-Au	immediate	
	BFO	2	

For Ag and Ag–Au nanocatalysts, the characteristic absorption peak of Ag at 419 nm remained, indicating the presence of Ag NPs in the solution.^31^ In the case of cationic dyes (MB and RhB), reversion is attributed to the instability of their reduced forms and subsequent reoxidation by atmospheric oxygen. However, this reoxidation is irreversible, as the reoxidized dye does not revert to its colorless form even upon agitation, like clock reactions. The reversal time varied among different nanocatalysts, and is likely influenced by catalytic efficiency, dye characteristics, the catalyst’s point of zero charge, and solution pH, all of which modulate the oxidation process.

To confirm that the reoxidation process is attributable to atmospheric oxygen rather than dissolved oxygen, typical of clock reaction, this study further investigates the reduction and oxidation behavior of MB under an inert atmosphere and in the presence of a strong oxidizing agent, such as hydrogen peroxide (H_2_O_2_).

#### 4.2.1 Role of Atmospheric Oxygen

Atmospheric oxygen plays a pivotal role in the redox reactions of MB, serving as the primary oxidizing agent responsible for reverting the reduced LMB form to its original oxidized state. Under nitrogen (N_2_) atmosphere, MB solutions containing all five nanocatalysts remained in the LMB form for over 24 h but reverted to MB upon exposure to air, confirming that atmospheric oxygen, rather than dissolved oxygen^31^ is the key oxidant (Figure 5).

**Figure 5:**
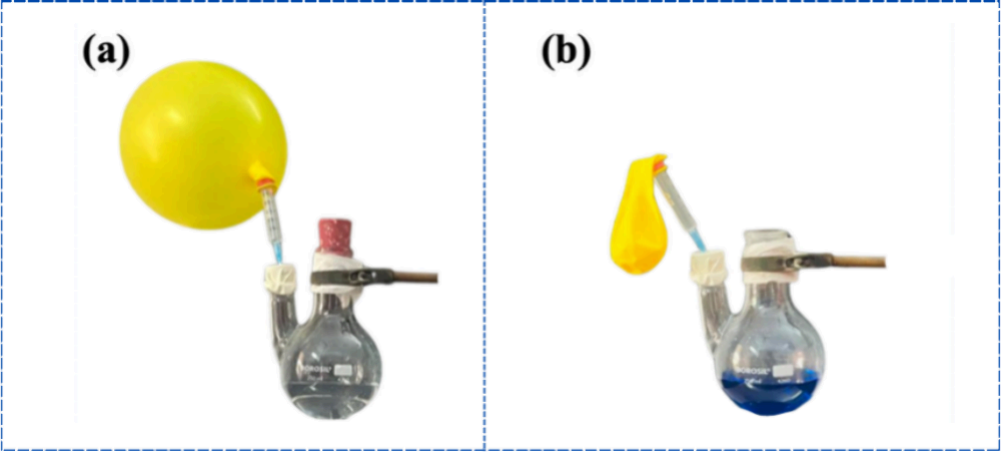
(a) Reduced LMB maintained under nitrogen (N_2_) atmosphere, preventing oxidation; (b) reversion of LMB back to colored MB upon exposure to air after N_2_ balloon deflation.

#### 4.2.2 Hydrogen Peroxide (H_2_O_2_) Effect

H_2_O_2_ serves as a potent oxidizing agent, facilitating the generation of hydroxyl radicals (•OH) and reactive oxygen species that enhance the oxidation of reduced substances.^29^ An experiment utilizing H_2_O_2_ in the absence of nanocatalysts to assess the role of hydroxyl ions in the reduction of MB revealed that, although H_2_O_2_ produces hydroxyl radicals via Fenton’s reaction, the presence of a catalyst is essential for the efficient execution of the redox process (Figure 6a). Nevertheless, in systems where a dye has been reduced to a colorless form, H_2_O_2_ swiftly reoxidizes the dye to its original-colored state by generating oxygen or hydroxyl radicals that facilitate the reverse reaction (Figure 6b,c).

**Figure 6:**
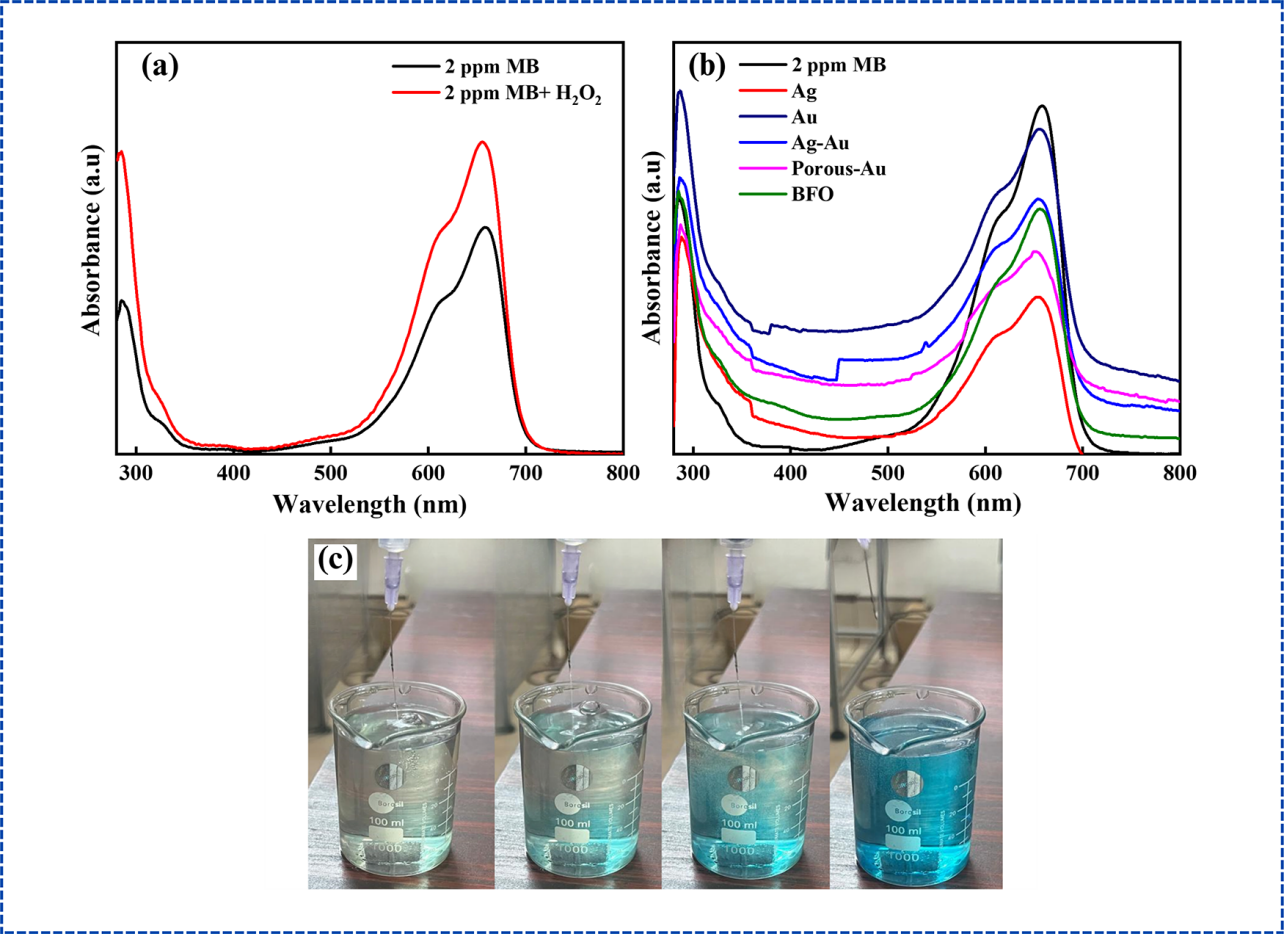
(a) Behavior of MB in the presence of H_2_O_2_ alone, (b) reoxidation of MB from LMB upon the addition of H_2_O_2_ to MB–NaBH_4_ mixtures in the presence of various nanocatalysts, and (c) real-time image of reoxidation from MB to LMB.

A similar trend was observed for RhB (Figure S4), whereas MO and CR remained colorless even after air exposure or the introduction of an oxidizing agent, confirming that anionic dyes undergo complete reduction.

Parameters such as initial dye concentration, catalyst concentration, and solution pH also influence dye decolorization; the effects of these parameters were systematically studied to understand their role in the reduction and reoxidation of MB in detail.

#### 4.2.3 Initial Dye Concentration

Experiments conducted with varying dye concentrations (1–10 ppm) revealed that all solutions decolorized instantly, except for Ag at 5 and 10 ppm, where no reduction was observed. The time required for LMB to revert to MB decreased with increasing dye concentration (Figure 7a). This behavior is attributed to the enhanced adsorption of LMB onto the nanocatalyst surface at higher concentrations, which facilitates interactions with oxygen species and accelerates reoxidation.^28^ The absence of reduction in Ag at higher concentrations may be due to the saturation of available adsorption sites at these elevated levels.^28^

**Figure 7:**
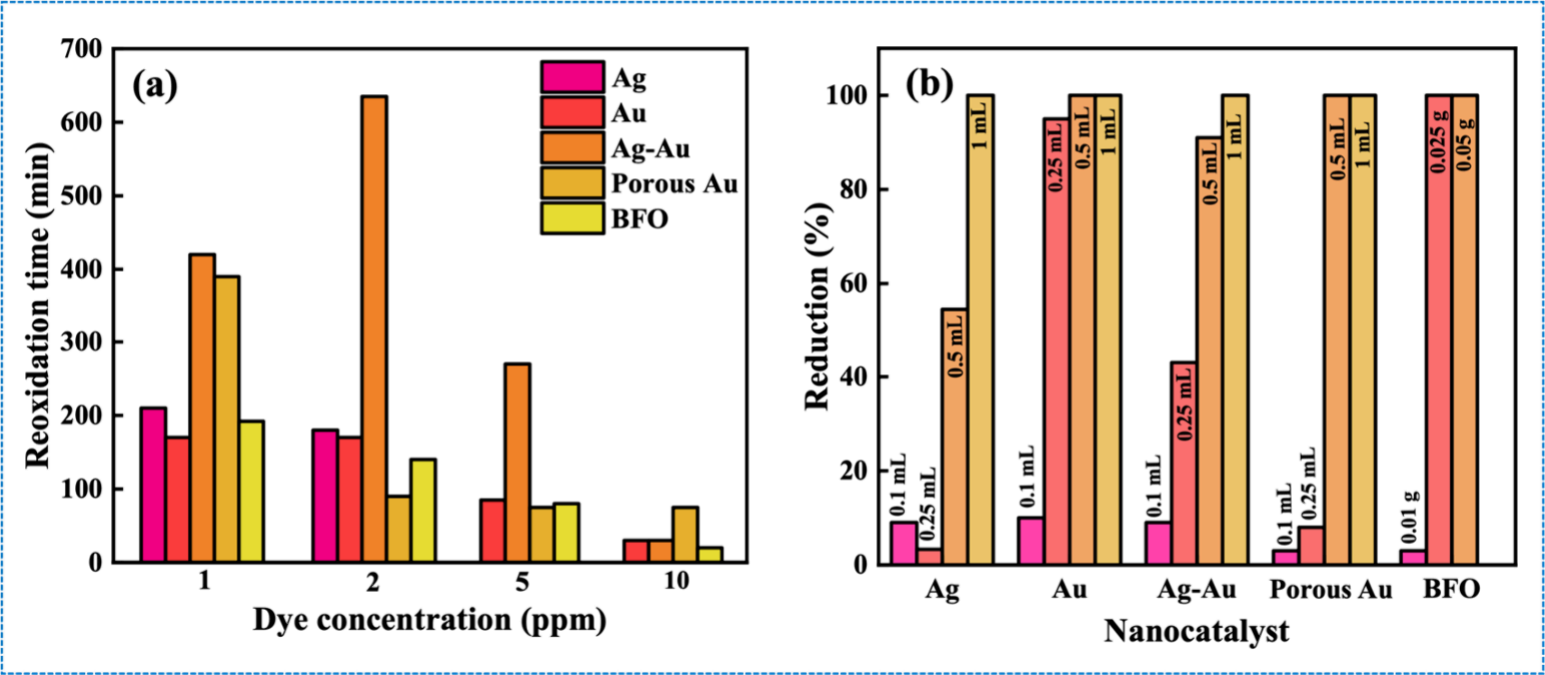
(a) Reoxidation time for various nanocatalysts at different dye concentrations and (b) reduction percentages of MB in the presence of varying concentrations of different nanocatalysts.

#### 4.2.4 Catalyst Concentration

The catalytic activity of colloidal porous Au NPs was evaluated by varying their volumes (1 mL, 2.5 mL, 5 mL, and 10 mL) in a mixture of MB and NaBH_4_ solution. No reduction was observed with 1 or 2.5 mL, whereas 5 mL resulted in immediate and complete reduction of MB to LMB (Figure 7b). The immediate reduction observed with 5 mL is attributed to the increased surface area, providing sufficient adsorption sites for the reaction. Increasing the catalyst volume to 10 mL showed no additional effect, indicating a saturation point, suggesting that the available adsorption sites had reached saturation. Similar trends were observed with other nanocatalysts. This indicates that increasing the nanocatalyst concentration improves reduction efficiency only up to a threshold, beyond which further increments become redundant.^28^

#### 4.2.5 pH Influence

The effect of pH on the catalytic reduction of organic dyes is a critical parameter influencing both the efficiency and mechanism of the reaction.^46–48^ In this study, the optimal pH range (4–13) for MB reduction was investigated by maintaining a constant MB concentration (2 ppm) and fixed levels of NaBH_4_ and nanocatalysts. The redox behavior varied significantly across catalysts and pH levels (Table 4). The enhanced catalytic performance of BFO NPs in basic media can be primarily attributed to its pH-dependent surface chemistry and defect-mediated activity. The point of zero charge (pH_PZC_) of BFO NPs has been reported to be ∼8.0, indicating that the surface remains positively charged under acidic conditions and transitions to a negatively charged state in alkaline environments.^49^ At pH values above the pH_PZC_, the negatively charged BFO NPs surface promotes strong electrostatic attraction toward cationic dye molecules (MB), thereby facilitating enhanced adsorption and subsequent reduction.^27,49^ This is consistent with the observed maximum degradation efficiency at basic pH, where increased surface negativity significantly strengthens dye–catalyst interactions through electrostatic forces.^49^ In addition, the interaction of NaBH_4_ with BFO NPs under basic conditions is likely to induce the formation of oxygen vacancies and surface defects, which serve as active catalytic sites and further enhance electron transfer efficiency.^27,47,48^ In contrast, under acidic conditions, surface protonation leads to competitive adsorption of H^+^ ions at active sites, thereby suppressing MB adsorption and significantly inhibiting the overall reduction efficiency.

**Table 4:** Reduction of MB at Different pH Levels in the Presence of Various Nanocatalysts (Note: *D* = Disappearance time; *R* = Reappearance time)

nanocatalyst	pH 4 (D/R)	pH 6 (D/R)	pH 7 (D/R)	pH 10 (D/R)	pH 13 (D/R)
Ag	2 min/40 min	2 min/40 min	2 min/40 min	8 min/2 h	no reduction
Au	1 min/2.3 h	1 min/2.3 h	1 min/4 h	no reduction	no reduction
Ag–Au	faded/2 min	faded/2 min	1 min/4 h	no reduction	no reduction
porous Au	faded/2 min	faded/2 min	1 min/1 h	1 min/4 h	no reduction
BFO	no reduction	no reduction	1 min/4 h	1 min/4 h	1 min/not returned

However, metal NPs, including Ag, Au, Ag–Au, and porous Au, performed better under acidic conditions compared to basic conditions, which may be attributed to the partial dissolution or activation of surface metal oxides on the NPs, thereby increasing the availability of catalytically active sites and accelerating the reduction.^50^ Furthermore, the reported zeta potential values suggests that the synthesized metal NPs may retain negative surface charges even under acidic conditions (Ag ≈ −25.8 mV, Au ≈ −27.4 mV, Ag–Au ≈ −24 mV, and porous-Au ≈ −38.5 mV).^41,51–53^ This could potentially enhance the electrostatic attraction between the negatively charged nanoparticle surfaces and cationic MB molecules, thereby facilitating improved adsorption and contributing to faster reduction kinetics.

All the aforementioned studies collectively support our observation that nanocatalyst-assisted dye degradation in the presence of NaBH_4_ exhibits distinct behavior for cationic and anionic dyes.

Previous studies on dye reoxidation have predominantly focused on MB, with no investigation extending to other dye systems. Reported findings indicate that atmospheric oxygen alone is often insufficient to reconvert LMB to its oxidized form; instead, external oxygen supply (e.g., ∼1 bar pressure) or alkaline conditions are required to facilitate the clock reaction. For instance, the use of NaOH in the presence of Cu nanowires has been shown to promote MB oxidation by generating hydroxyl ions and creating an oxygen-rich environment conducive to reoxidation.^17^ In contrast, another study attributed the apparent recovery of MB color not to true oxidative cycling, but to the progressive decomposition of NaBH_4_, which diminishes the availability of electrons required for sustained reduction. As NaBH_4_ degrades over time, the reduction rate decreases, thereby enabling the reoxidation of LMB by dissolved oxygen.^31^ However, this investigation was limited to Ag NPs synthesized via different methods.

Despite these insights, existing literature remains limited to cationic dyes such as MB, with no systematic comparison involving anionic dyes. Notably, while NaBH_4_ degradation has been proposed as the primary driver for reoxidation in MB systems, our observations reveal that such reversible behavior is absent in anionic dyes, suggesting that the underlying mechanism is not solely governed by reductant stability but is intrinsically linked to the molecular structure and bonding characteristics of the dye. This distinction highlights a critical gap in current understanding.

Therefore, to elucidate the mechanistic differences between cationic and anionic dye systems, we provide a plausible mechanism for NaBH_4_-mediated reduction, supported by FTIR analysis that probes structural and functional group transformations during the reaction, thereby revealing the nature of bond alterations that account for the distinct degradation pathways where cationic dyes remain susceptible to reoxidation, while anionic dyes undergo irreversible degradation.

### 4.3 Plausible Mechanism for the Reduction of Dye in the Presence of NaBH_4_

It is well-known that the reduction of organic dyes using NaBH_4_ in the presence of nanocatalysts is governed by a surface-mediated electron relay mechanism involving both physicochemical interactions and catalytic processes. Initially, dye molecules are adsorbed onto the catalyst surface through intermolecular interactions, followed by the adsorption of BH_4_^–^ ions generated from the dissociation of NaBH_4_ in solution.^26^ These BH_4_^–^ ions act as strong electron donors, transferring electrons to the catalyst surface, which subsequently mediates electron transfer to the dye molecules, resulting in their reduction to colorless forms. Concurrently, the interaction of BH_4_^–^with water and the nanocatalyst surface leads to the generation of reactive hydrogen species, which play a crucial role in the hydrogenation of dye molecules. The reaction can be described as follows:

$${\mathrm{BH}}_{4}^{-}+2H_{2}O\to {\mathrm{BO}}_{2}^{-}+4H_{2}$$

$${\mathrm{BH}}_{4}^{-}+4{\left[\mathrm{Dye}\right]}^{+}+2H_{2}O\to {\mathrm{BO}}_{2}^{-}+4\left[\mathrm{Reduced}\;\mathrm{Dye}\right]+4H^{+}$$

Nanocatalysts function as efficient electron relays, facilitating rapid electron transfer from BH_4_^–^ to the adsorbed dye molecules, thereby significantly enhancing the reaction kinetics.^29,31,54^ Furthermore, the adsorption of BH_4_^–^ ions onto the catalyst surface induces the formation of a negatively charged layer, which promotes the electrostatic attraction of cationic dyes such as MB and RhB, thereby improving adsorption and accelerating reduction rates.^17^

The chemical transformations and corresponding functional groups involved in the reduction process are illustrated in Figure 8. It is well established that MB, a cationic thiazine dye, dissociates in aqueous solution into chloride ions and positively charged dye species, with its cationic character arising from the quaternary ammonium group (-NR_4_^+^).^27,47^ During the reduction process, NaBH_4_ acts as a hydrogen donor, facilitating the transfer of electrons and reactive hydrogen species to the aromatic framework of MB. This leads to the disruption of the conjugated π-electron system within the phenothiazine ring, resulting in the formation of its reduced, colorless LMB form.^55^ Mechanistically, the transferred electrons, in conjunction with activated hydrogen species, attack the chromophoric functional groups (e.g., – C=N−), inducing hydrogenation (N–H) bond formation and loss of color.^26^ The evolution of hydrogen gas, typically observed as bubble formation, further supports the role of BH_4_^–^ as both an electron donor and a hydrogen source in the reaction. However, the reduced LMB form is unstable and readily reoxidizes to MB upon exposure to atmospheric oxygen; consequently, the N–H bond formed during reduction cleaves, demonstrating the reversible nature of the system (Figure 8a). Similarly, RhB, a cationic xanthene dye, undergoes reduction through hydrogenation of the imine (C=N) group, converting the electrophilic C=N bond into a secondary amine (CH–NH) via hydride attack and subsequent protonation.^56–59^ Similar to MB, this reduction process is reversible, as atmospheric oxygen promotes reoxidation accompanied by cleavage of the N–H bond (Figure S5). In contrast, for anionic dyes such as MO and CR, the azo group (−N=N−) undergoes successive hydrogenation to form intermediate −NH–NH– species, which subsequently undergo bond cleavage. This irreversible cleavage of the azo linkage prevents reoxidation to the parent dye, thereby resulting in permanent degradation of the dye molecules. This transformation highlights the role of nanocatalysts in converting hazardous azo dyes into safer amine products (Figure 8b and Figure S6).^60–65^ From this discussion, it is evident that cationic dyes undergo reduction but are prone to reoxidation due to interaction with atmospheric oxygen, whereas anionic dyes, upon reduction, are irreversibly converted into nontoxic derivatives.

**Figure 8:**
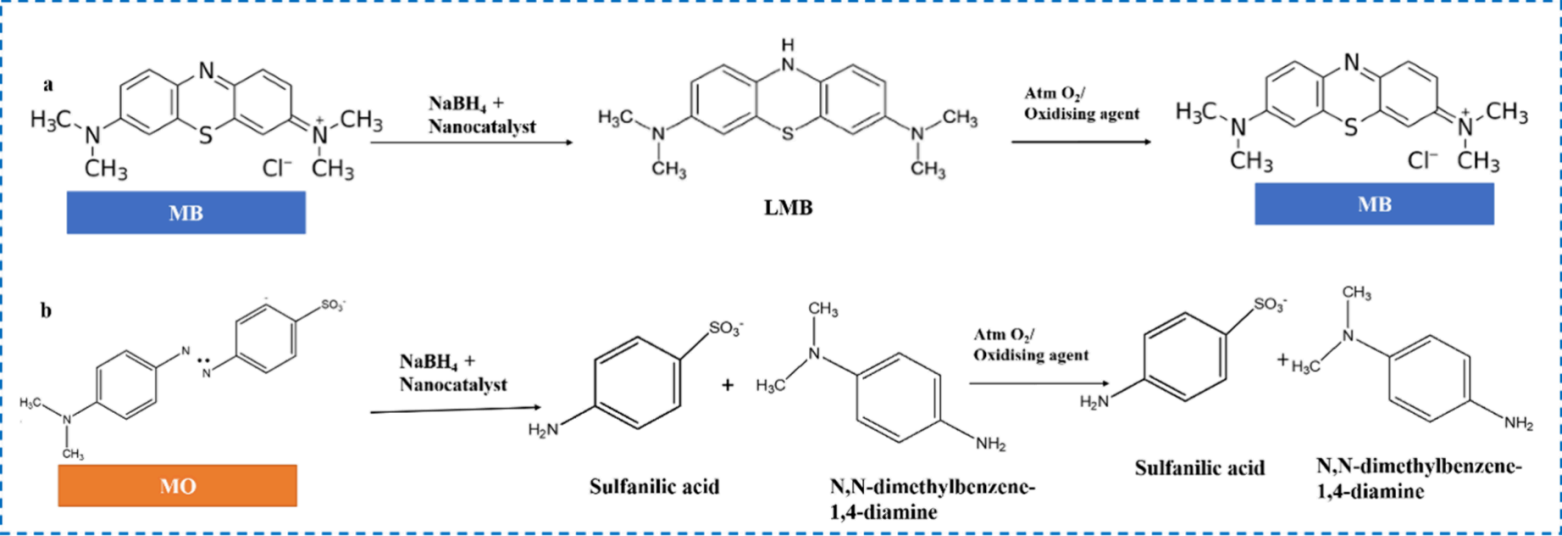
Reduction behavior of (a) MB and (b) MO in the presence of NaBH_4_.

### 4.4 FTIR Spectroscopy

FTIR spectroscopy of MB (C_16_H_18_N_3_ClS) in its natural state (pristine), reduced (LMB, C_16_H_19_N_3_S ^–^), and reoxidized forms (Figure 9) revealed characteristic peaks for C–S–C stretching (619 cm^–1^), C–N stretching (1117 cm^–1^), C–N stretching (1344 cm^–1^), and C–H bending (1398 cm^–1^), and N–H bending (1630 cm^–1^) in all three forms. However, the appearance of a small N–H bending peak at 1402 cm^–1^ along with a broad N–H stretching band at 3478 cm^–1^, observed exclusively in the reduced form, confirms the formation of N–H bonds.^66,67^ This indicates that NaBH_4_, acting as a hydride donor, reduces MB by transferring hydrogen to its aromatic structure, disrupting the conjugated system of the phenothiazine ring and converting MB to its colorless LMB form. The presence of covalently bonded hydrogen in LMB, as evidenced by the N–H peak,^55^ which is absent in both pristine and reoxidized forms, further demonstrates the reversible conversion of LMB to MB over time.

**Figure 9:**
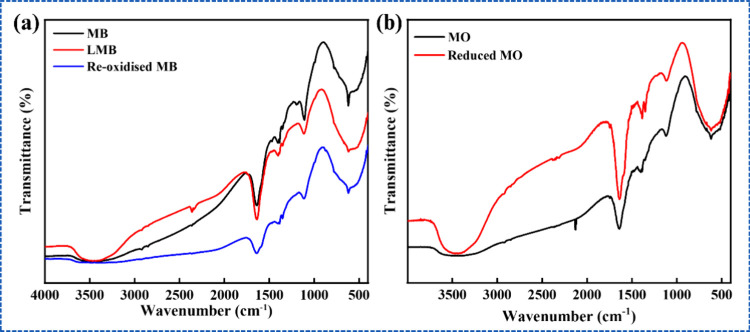
FTIR spectra for (a) MB, LMB, reoxidized MB and (b) MO, reduced MO.

Similar to MB, FTIR analysis was conducted on RhB to confirm the reoxidation process by examining its spectra before reduction, after reduction, and following reoxidation (Figure S7). Key peaks observed in all three samples included 633 cm^–1^ (C–H bending), 1117 cm^–1^ (C–N vibrations), 1349 cm^–1^ and 1388 cm^–1^ (C–N and C–H stretching of aromatic amine), and 1634 cm^–1^ (C=N stretching of imine group), along with a minor peak at 2365 cm^–1^ attributed to CO_2_ adsorption.^59,66,68^ A prominent peak at 3459 cm^–1^, corresponding to N–H stretching in secondary amines,^66^ was detected exclusively in the reduced RhB, confirming the reduction mediated by NaBH_4_. The absence of this N–H peak in the spectra of both pure and reoxidized RhB validated the reversibility of the reduction process.

The reduction of RhB (C_28_H_31_ClN_2_O_3_) by NaBH_4_ involves hydrogenation of the imine group (C=N) to form Leuco-Rhodamine B (C_28_H_33_ClN_2_O_3_), a colorless derivative. This transformation occurs via hydride ions (H^–^) generated by NaBH_4_ in aqueous media, which reduce the electrophilic C=N bond to a secondary amine (CH–NH) through hydride attack and subsequent protonation. This process disrupts the conjugated chromophore system of RhB, leading to decolorization.^58^ The reappearance of the original FTIR peaks upon reoxidation further demonstrates the reversible nature of this chemical transformation.

The FTIR spectra of MO (C_14_H_14_N_3_NaO_3_S) dye solution before and after reduction (Figure 9b), revealed characteristic peaks at 619 cm^–1^, corresponding to aromatic out-of-plane C–H bending of the benzene ring; 1107 cm^–1^, attributed to −C–N stretching vibrations; 1383 cm^–1^, confirming the S=O stretching vibrations indicative of the sulfonic nature of MO; and 1639 cm^–1^, associated with C=C bond vibrations or C=N stretching within the MO structure.^61,66^ The band observed at 1330 cm^–1^ in reduced MO is attributed to vibrations associated with the sulfonate group, which are not clearly observed in pure MO, possibly due to the relatively weaker bond environment or lower intensity in the pristine structure. Furthermore, the characteristic azo (−N=N−) stretching vibration becomes significantly weakened or nearly absent in both MO and reduced MO spectrum. This reduction in band intensity can be attributed to the low polarity and increased molecular symmetry of the reduced intermediate. In addition, an additional broad band at 3473 cm^–1^ appears in the reduced MO spectrum, corresponding to N–H stretching vibrations of amine groups, which provides strong evidence for the reduction of the azo linkage to an amine-type intermediate (−NH–NH−). This transformation leads to the formation of a colorless intermediate under the given reaction conditions.^60–62,65^

The FTIR spectra of CR dye and its reduced counterpart reveal distinct molecular signatures that clearly delineate the structural transformations occurring during the reduction process. Characteristic bands observed at 2000 cm^–1^ are attributed to aromatic C–H stretching vibrations, while the broad band at 2550 cm^–1^ is assigned to O–H stretching. The weak bands observed at 2142 and 2278 cm^–1^ are not conclusively assigned and may arise from atmospheric CO_2_ adsorption or possible intermediate species (Figure S8).^66,69,70^ Additionally, the band around 2340 cm^–1^ is attributed to CO_2_ adsorption.^66^ A prominent absorption band at ∼1600 cm^–1^, assigned to the azo (−N=N−) linkage, disappears upon reduction, providing direct evidence for azo bond cleavage.^69^ Concurrently, the emergence of a band at 1270 cm^–1^, associated with S=O stretching, confirms the persistence of sulfonate functional groups in the reduced products.^69^ The presence of a broad band around 3350 cm^–1^, corresponding to N–H stretching, in both spectra suggests partial hydrogenation during the reduction process.^66,69,70^ Collectively, these spectral changes substantiate the effective degradation of CR into its reduced form.

Mechanistically, the azo (−N=N−) group of CR undergoes stepwise hydrogenation in the presence of NaBH_4_, initially forming a hydrogenated azo intermediate (−NH–NH−). Further hydrogenation induces cleavage of this intermediate, yielding amine derivatives such as biphenyl and sodium 4-amino-1-naphthalenesulfonate. Importantly, these transformation products are comparatively less toxic than the parent azo compound, underscoring the environmental significance of the reduction pathway.^62,71^

Therefore, the nature and distribution of functional groups within dye molecules critically dictate the decolorization pathway, governing not only the reduction kinetics but also the propensity for reoxidation and the overall stability of the system. These structural determinants ultimately define the reversibility of the process, distinguishing cationic dyes, which are prone to reoxidation, from anionic dyes, which undergo irreversible bond cleavage and persistent degradation, thereby establishing a clear structure–reactivity relationship in the decolorization mechanism.

### 4.5 Recyclability

The recyclability of a catalyst is a critical parameter for evaluating its practical applicability. In the present study, the recyclability of BFO NPs was assessed over five consecutive cycles under identical experimental conditions for both Mb and MO. After each cycle, the catalyst was separated from the reaction mixture via centrifugation, followed by thorough washing and drying prior to reuse. The reduction efficiency remained constant for MB (100%), while in the case of MO it decreased only marginally from 97.5% in the first cycle to 97.3% in the fifth cycle (Figure S9), indicating negligible loss of catalytic activity and good stability of the BFO NPs catalyst. In contrast, recyclability studies could not be performed for the other four catalysts (Ag, Au, Ag–Au, and porous Au NPs), as they were employed in colloidal form and could not be effectively recovered from the dye solution under the present experimental conditions.

## 5 Conclusions

The reduction of various organic dyes, including cationic dyes like MB and RhB, and anionic dyes such as MO and CR, using NaBH_4_ was systematically examined using UV–vis spectroscopy and FTIR. While NaBH_4_ exhibited strong reducing power, it was insufficient for complete dye reduction without nanocatalysts. The addition of Ag, Au, Ag–Au, porous Au, and BFO NPs significantly enhanced the reduction process. Notably, cationic dyes underwent reversible reduction, with reoxidation depending on catalyst efficiency, whereas anionic dyes exhibited irreversible reduction.

The role of environmental factors in MB reduction was examined under nitrogen, atmospheric oxygen, and H_2_O_2_, highlighting their influence on reversibility. Reduction efficiency increased with nanocatalyst concentration until saturation, while higher dye concentrations accelerated LMB reoxidation via enhanced adsorption and oxygen-mediated oxidation. The catalytic performance varied with pH, where BFO NPs were more effective under alkaline conditions due to defect generation, while metal NPs performed optimally in acidic media due to enhanced electron transfer. FTIR analysis of the original, reduced, and reoxidized dye forms corroborates these findings, confirming the role of NaBH_4_ as an efficient hydride donor in the reduction process. Importantly, the nature of the functional groups inherent to the dye structure plays a role in dictating the reduction pathway and subsequent redox behavior. In cationic dyes, functional groups such as heterocyclic nitrogen centers facilitate reversible reduction through the formation of unstable intermediates, enabling reoxidation upon exposure to atmospheric oxygen. In contrast, anionic dyes containing azo (−N=N−) linkages undergo irreversible hydrogenation and bond cleavage, leading to stable amine derivatives that resist reoxidation. This distinction underscores the critical influence of functional group chemistry in governing the differential redox responses of cationic and anionic dye systems.

## Supplementary Material



## Data Availability

The data is available throughout the manuscript and supporting files.

## Notes

UV–vis absorbance evolution of porous Au nanoparticles; reduction of MB, RhB, MO, and CR in the presence of NaBH_4_; reduction and reoxidation behavior of CR and RhB with different nanocatalysts; FTIR spectra of dyes before and after reduction; recyclability studies of MB and MO in the NaBH_4_–BFO system (PDF)
