# Supplementary material for: Reductive Stability of Organic Dyes in Nanocatalyst-Assisted Sodium Borohydride Systems
Source: ACS Omega. 2026 May 5;11(19):28569–81. doi: 10.1021/acsomega.6c00189 (PMC13191550; doi:10.1021/acsomega.6c00189)
Supplement: Supplementary file 1 [file ao6c00189_si_001.pdf]

Reductive Stability of Organic Dyes in Nanocatalyst-Assisted Sodium Borohydride Systems

Rahina Mohammad Kunhi[1], Jishina Karunakaran[1†], Rashmi Kunhiraman[1†], Manjunatha Pattabi[1], Nanditha Thayyath Kizhakkeveettil[2], Rani Manjunatha Pattabi[1*], Gurumurthy Sangam Chandrasekhar[2*]

[1] Department of Materials Science, Mangalore University, Mangalagangotri-574199, India.

[2] Manipal Institute of Technology, Manipal Academy of Higher Education, Manipal, Karnataka-576104, India

†These authors contributed equally to this work

*Corresponding authors: ranimpattabi@rediffmail.com, gurumurthy.sc@manipal.edu

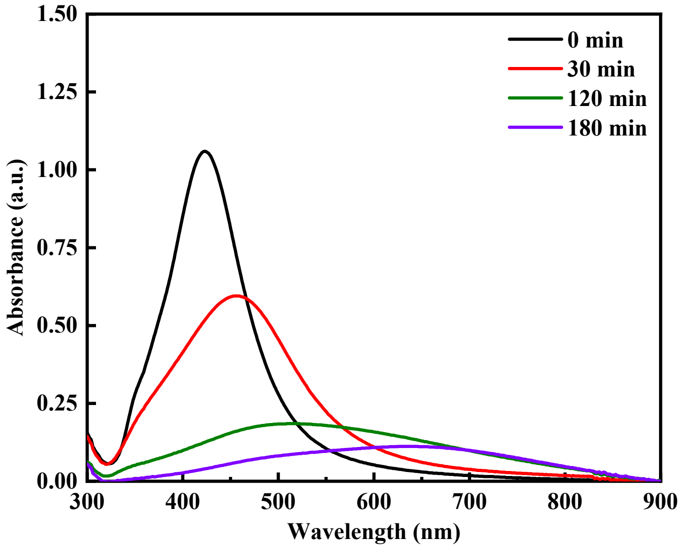

Fig. S1. Evolution of UV-Absorbance spectra of Au porous nanoparticles with time.

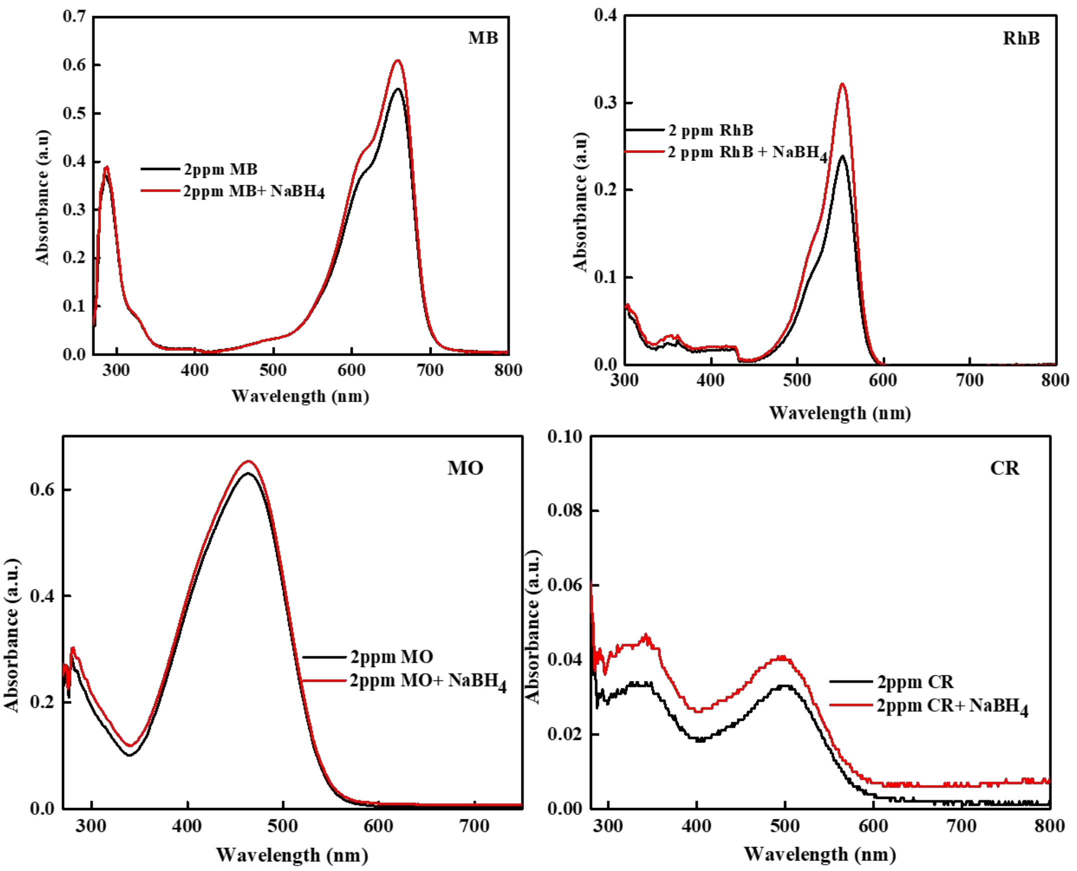

Fig. S2. Reduction of MB, RhB, MO and CR in presence of $NaBH_4$ alone

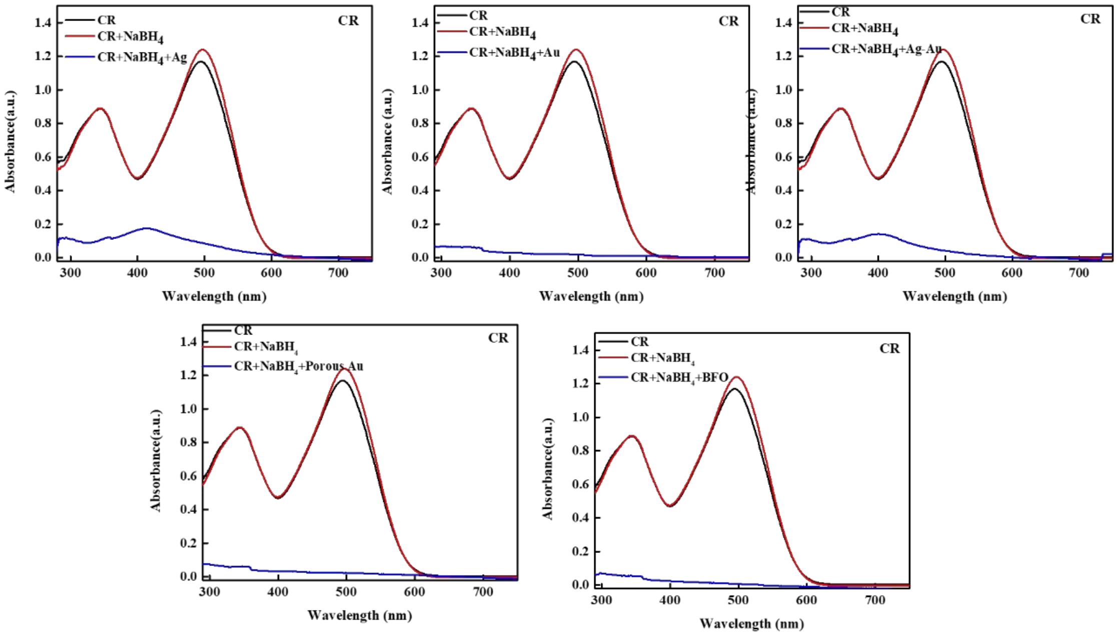

Fig. S3. Reduction and reoxidation of CR in the presence of $NaBH_4$ and various nanocatalysts, including Ag, Au, Ag–Au, porous Au, and BFO, under ambient conditions.

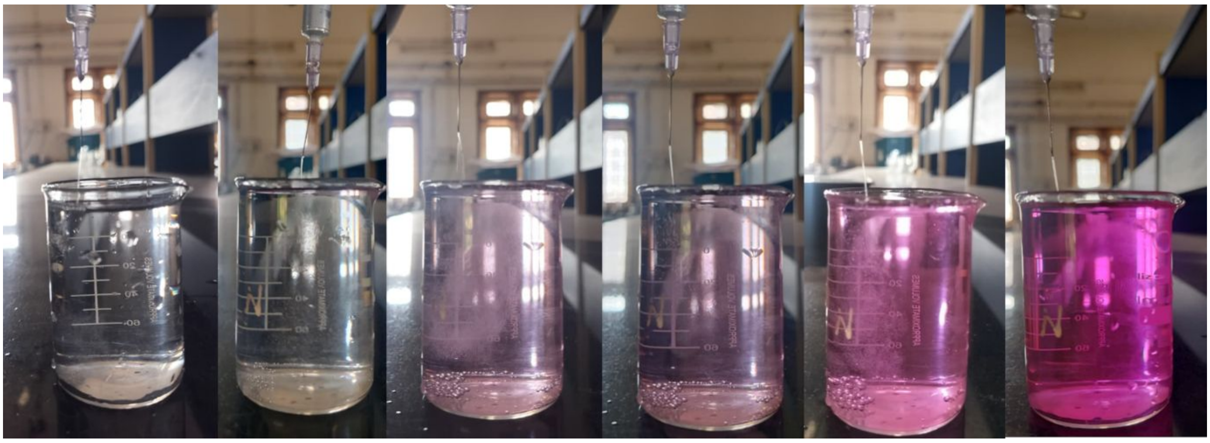

Fig. S4. Reoxidation of RhB from reduced RhB upon the addition of H_2O_2 to RhB–$NaBH_4$ mixtures in the presence of various nanocatalysts.

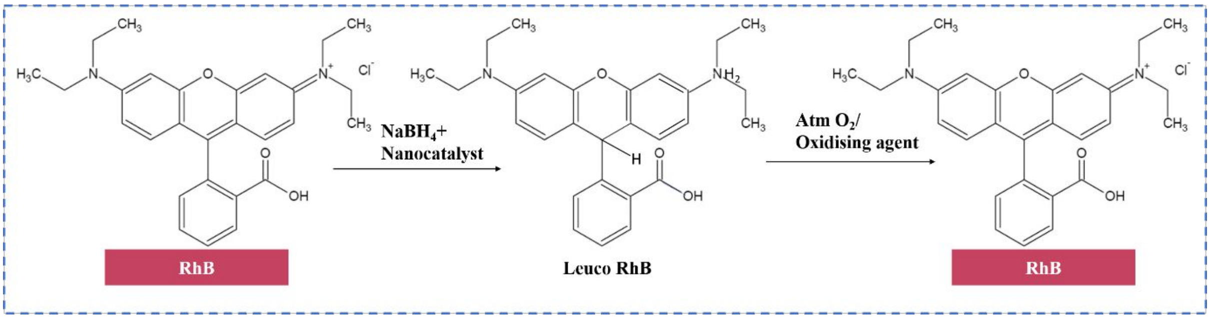

Fig. S5. Reduction behaviour of RhB, in the presence of $NaBH_4$.

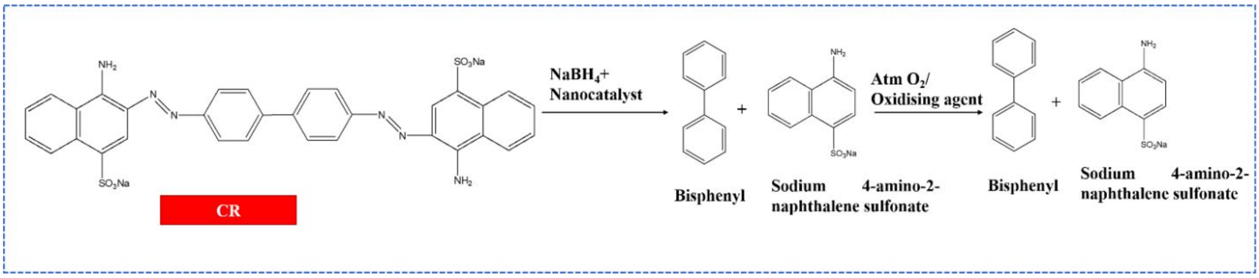

Fig. S6. Reduction behaviour of CR in the presence of $NaBH_4$.

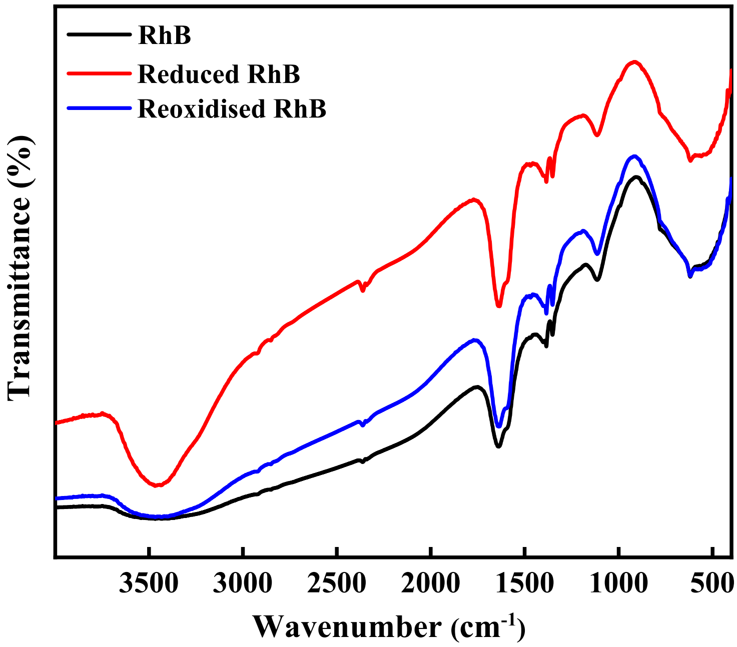

Fig. S7. FTIR spectra for RhB, Reduced RhB, and Reoxidised RhB.

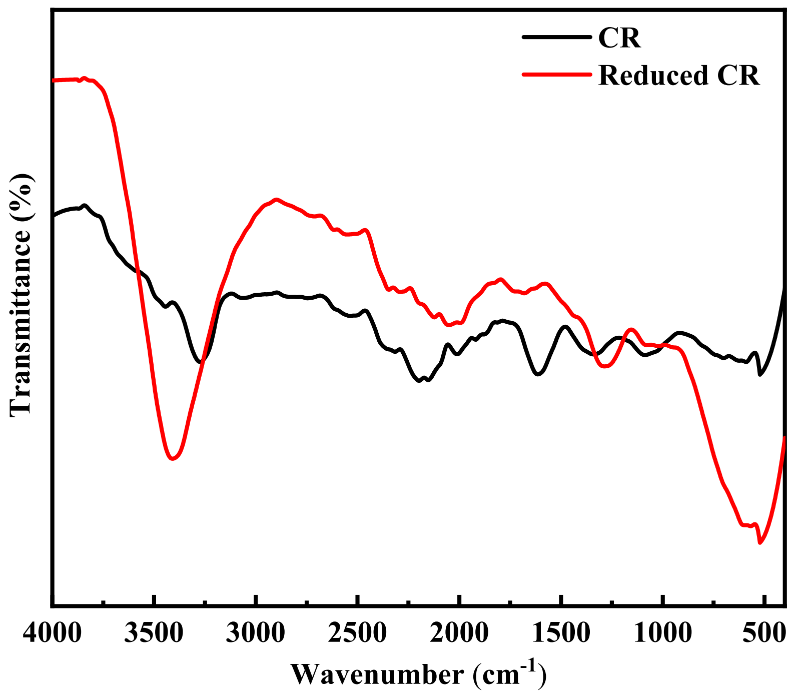

Fig. S8. FTIR spectra for CR and Reduced CR.

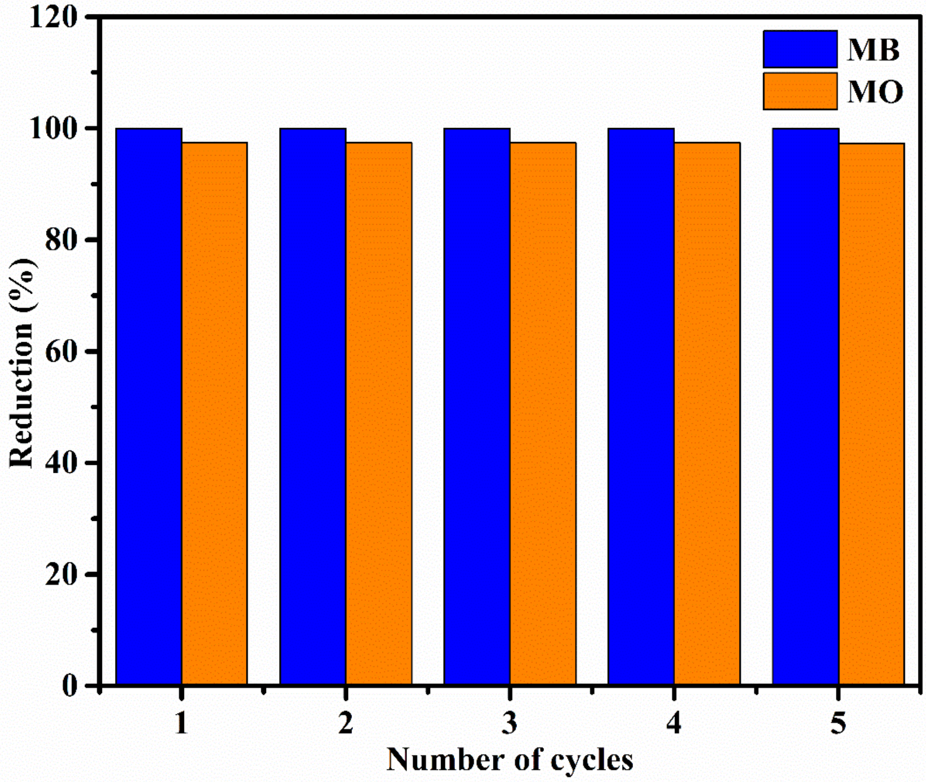

Fig. S9. Recyclability of MB and MO in $NaBH_4$- BFO system
